# Epigenetic Priming in Childhood Acute Lymphoblastic Leukemia

**DOI:** 10.3389/fcell.2019.00137

**Published:** 2019-07-17

**Authors:** Javier Raboso-Gallego, Ana Casado-García, Marta Isidro-Hernández, Carolina Vicente-Dueñas

**Affiliations:** ^1^Experimental Therapeutics and Translational Oncology Program, Instituto de Biología Molecular y Celular del Cáncer, CSIC/Universidad de Salamanca, Salamanca, Spain; ^2^Institute of Biomedical Research of Salamanca (IBSAL), Salamanca, Spain

**Keywords:** epigenetic priming, reprogramming, childhood leukemia, B-ALL, T-ALL, stem cells

## Abstract

Leukemogenesis is considered to be a process by which a normal cell acquires new but aberrant identity in order to disseminate a malignant clonal population. Under this setting, the phenotype of the leukemic cells is identical to the leukemia-initiating cell in which the genetic insult is taking place. Thus, with some exceptions, B-cell and T-cell childhood leukemias are supposed to arise from B- or T-committed cells. In contrast, several recent studies have revealed that genetic alterations may act in a “hit-and-run” way in the cell-of-origin by imposing the tumor cell identity giving rise to either B-cell or T-cell leukemias. This novel mechanism of cell transformation is mediated by an epigenetic priming mechanism that is established by the initial genetic lesion. This initial hit might be unnecessary for the subsequent tumor evolution and conservation, being the epigenetic priming the engine for the tumor evolution.

## Childhood Acute Lymphoblastic Leukaemia (ALL)

Childhood Acute lymphoblastic leukaemia (ALL) is the most common malignancy in children (Hunger and Mullighan, 2015). Childhood ALL is composed of two main immunophenotypes that are identified by distinctive hematopoietic lineage markers: B- and T-ALL. Approximately, 85% of ALL cases are paediatric B-ALL while T-ALL comprises the remaining 15% (Hunger and Mullighan, 2015).

Cytogenetic analyses have revealed that persistent structural and copy number genomic aberrations are hallmarks of ALL (Iacobucci and Mullighan, 2017; Pui et al., 2019b). Cytogenomic subtyping usually occurs through visual chromosome analysis by the use of fluorescence *in situ* hybridization (FISH), karyotyping via G-banding, and/or chromosomal microarray (Mrozek et al., 2009).

Currently, up to 90% of childhood ALL patients can be categorized into distinct genetic subtypes (Pui et al., 2019a), the prognostic characteristics and treatment response differ among them (Roberts and Mullighan, 2015; Iacobucci and Mullighan, 2017).

The genetic alterations are specific of each ALL immunophenotype being for examples hyperdiploidy (50–67 chromosomes); hypodiploidy (44 chromosomes or fewer); *BCR-ABL1, ETV6-RUNX1*, or *TCF3-PBX1* fusions; *PAX5* or *ETV6* mutations, MLL rearrangements, or intrachromosomal amplification of chromosome 21 (iAMP21) specific for B-ALL, whilst alterations in *LMO2, TAL1, TAL2, TLX1, TLX2*, or *HOXA* are characteristics of T-ALL (Pui et al., 2019a).

A newly revised taxonomy of B-ALL incorporates 23 subtypes well-defined by chromosomal rearrangements, sequence mutations or heterogeneous genomic changes (Gu et al., 2019), highlighting the genetic heterogeneity of this disease. Most of these molecular changes are acquired, not inherited, as only a small number of leukaemias are associated with inherited genetic syndromes as is the case for germline mutations in *PAX5* (Auer et al., 2014; Martin-Lorenzo et al., 2018), *ETV6* (Moriyama et al., 2015; Noetzli et al., 2015) or *IKZF1* (Churchman et al., 2018).

With few exceptions, it is thought that childhood ALL is the result of the aberrant transformation of progenitor B- or T-cells in the bone marrow into leukemic cells giving rise to B- or T-ALL. This could be in line with the fact that the genetic alterations are only present in one immunophenotype and do not share in both. However, the mechanism triggering this leukemic conversion is not well comprehended and the cell-of-origin or the leukemia-initiating cell (LIC) seems not to be necessarily a committed B- or a T-cell (Rodriguez-Hernandez et al., 2017; Garcia-Ramirez et al., 2018; Martin-Lorenzo et al., 2018). Likewise, it has been recently described for MLL-AF4+ infant B-ALL and TCF3-ZNF384+ B-ALL which both have a fetal liver hematopoietic stem cell as the cell-of-origin (Agraz-Doblas et al., 2019; Bueno et al., 2019).

Evidence from twin studies and revisions of neonatal blood spots suggest that most initiating events (“first hit”) occur during fetal development in utero (Ford et al., 1993; Gale et al., 1997; Wiemels et al., 1999; Greaves et al., 2003; Alpar et al., 2015; Bateman et al., 2015; Schafer et al., 2018; Bueno et al., 2019). But not all the preleukemic carriers will develop the disease, suggesting that a secondary event (“hit”) needs to take place in the preleukemic cell to fully transform it into a leukemic one. Under this scenario, the cancer cell-of-origin would be the initially healthy cell that will be primed by an oncogenic hit and that will give rise to a preleukemic cell. And the LIC would be the preleukemic cell that has acquired secondary mutations, and as a result, it promotes the complete transformation of the preleukemic clone. Within the context of the so-called “two-hit hypothesis,” the childhood ALL cell-of-origin and the LIC is still the focus of ongoing debate. The main problem that difficults to solve this issue is that human leukemia is diagnosed in the final stages of the transformation process when the LIC is fully converted into the leukemic one. For this reason, the identification of the cell-of-origin and the LIC, and the monitoring of the natural evolution of the disease from the first stages of the leukemia onset is almost impossible in humans (Cobaleda and Sanchez-Garcia, 2009).

For chromosomal translocations, like the ones found in lymphoid tumors, the chromosome breaks are usually caused by either AID or the RAG complex (Mahowald et al., 2008; Nussenzweig and Nussenzweig, 2010; Gostissa et al., 2011). As a matter of fact, Rag-1 expression has been detected in early progenitors in both mice and humans (Boiers et al., 2013, 2018). This early immune-restricted progenitor co-expressed myeloid and lymphoid lineage programs and contributed to both myeloid and lymphoid lineages in the embryo. Rag-1, therefore, provides a mechanistic possibility for translocations to happen at very early hematopoietic developmental stages in ALL. Thus, the cell-of-origin that suffers the initial hit of transformation (eg. Chromosomal translocation) will have the potential of transformation into a myeloid or lymphoid committed cell. But as there is genotype-phenotype correlation between the genetic alteration and the tumor phenotype in ALL, it seems to be reasonable that the genetic insult is setting the tumor phenotype in the uncommitted cell-of-origin.

Besides aberrant genetic modifications, epigenetic irregularities have been shown to trigger the pathogenesis of childhood ALL (Taylor et al., 2007; Chaber et al., 2017). It has been described that aberrant methylation is associated with prognosis (Wong et al., 2000), cytogenetic subtype (Zheng et al., 2004), prediction of treatment outcome (Milani et al., 2010) and relapse (Matsushita et al., 2004; Hogan et al., 2011) in childhood acute leukemias. And also, most different cytogenetic subtypes of ALL share recurrent DNA methylation changes presumably because of those changes are involved in their pathogenesis (Wong et al., 2012). In this sense, aberrant epigenetic modifications (DNA methylation, histone modification, and microRNA alterations) have been extensively reviewed elsewhere (Burke and Bhatla, 2014; Nordlund and Syvanen, 2018), but the focus of the present review is to pay attention to the epigenetic modifications taking place at the beginning of the transformation process, being the epigenetic change the step that leads to the preleukemic stage establishment.

## Epigenetic Priming as Cell Transformation Process

**FIGURE 1:**
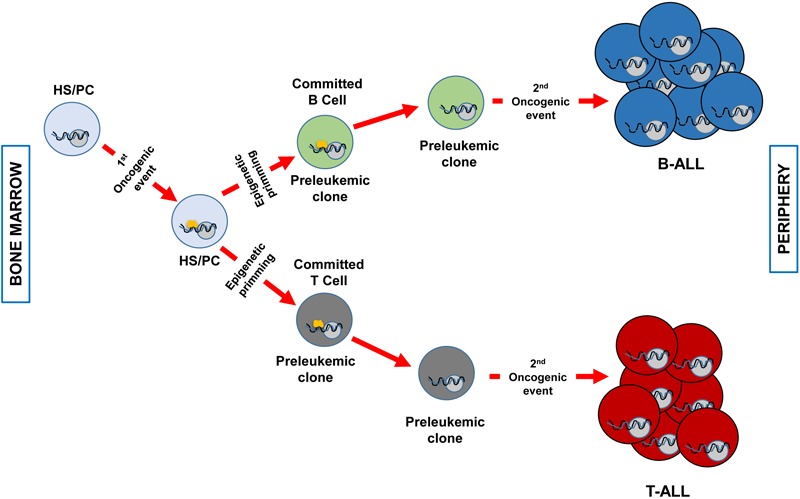
Clonal evolution of B and T-ALL imposed by epigenetic priming in genetically predisposed mouse models. The first genetic event that takes place in an HS/PC induces specific epigenetic priming that will determine the commitment of the preleukemic clone phenotype. For example, *ETV6-RUNX1* (Rodriguez-Hernandez et al., 2017) or *BCR-ABLp190* (Martin-Lorenzo et al., 2018) expression will prime a B-cell commitment, whilst the overexpression of *LMO2* will command T-cell priming (Garcia-Ramirez et al., 2018). Afterward, a second hit will transform the preleukemic clone in a fully transformed leukemic cell (B o T). At this stage, the first genetic event (represented in yellow) is not required anymore and has already primed the cell-of-origin into a specific differentiation cell program. A less efficient epigenetic priming could also occur in a more differentiated cell compartment as it has been described for LMO2 in T-ALL (Garcia-Ramirez et al., 2018).

There is a new accepted explanation that clarifies the connection between the specific immunophenotype in childhood ALL and the specific genetic disorder provoking it. As we have mentioned before, there are B- and T-ALL specific genetic lesions that are not shared between these two biological entities, there is a strong genotype-phenotype association. It has been traditionally thought that the driving genetic event was taking place in a committed B- or T-cell and this was the reason for the immunophenotype correlation. In recent years, it has been proposed that the genetic insult has the capacity to induce genetic priming in the cell-of-origin that imposes the ultimate phenotype of the transformed cell. This last option highlights a different role for the oncogenic hit. In this new concept of transformation mechanism, the first aberrant genetic insult is imposing a specific cell-differentiation program in the cancer cell-of-origin that will define the later phenotype of the tumor cells (for instance, being a B- or a T-cell). But of note, is that the initial oncogenic alteration essential for tumor initiation will not be necessary upon the differentiation program is started. So, in the later stages of transformation, the oncogene is not required and for this reason, it is not a good target for therapies because it does not have a critical role during tumor progression (Figure 1).

This also fits with the fact that specific epigenomic profiles can distinguish between cell lineage differentiation stages in the hematopoietic system and also in leukemic cells (Hu and Shilatifard, 2016; Carrillo-de-Santa-Pau et al., 2017). This epigenetic reprogramming has been proved in several animal cancer models that resemble human disease quite well. Conducting genome-wide DNA methylation profiling in these models [*MafB* in multiple myeloma (MM) (Vicente-Duenas et al., 2012), *Bcl6* in Diffuse Large B cell Lymphoma (DLBCL) (Green et al., 2014), *BCR-ABLp210* in chronic myeloid leukemia (CML) (Perez-Caro et al., 2009)] it has been identified a molecular reprogramming profile in the tumor-initiating cells (the hematopoietic stem/progenitor cells (HS/PC)), where the oncogene was expressed, that was still present in the tumor cells where the oncogene was switched off. In these models, the oncogene function in a “hit-and-run” way that works at an initial step of cell development to reprogram HS/PCs for malignancy. This epigenetic memory would maintain gene expression profiles throughout several cell generations, but not including modifications in the DNA sequence of the cells and in the absence of the initiating drivers of cell transformation, the expression of the oncogene. Interestingly, this tumoral epigenetic rewiring in progenitors cells is not limited to the hematopoietic system, but it is also a shared process that likewise has a role in non-hematopoietic tumors and has been reviewed elsewhere (Vicente-Duenas et al., 2018).

## Epigenetic Priming in Childhood B-ALL

B-ALL is a clonal malignant disease that initiates in a single cell and is characterized by an enlargement of precursor B-cells that are phenotypically similar to healthy precursors B-cells. This is why it has been argued that a committed B cell should be the B-ALL cell-of-origin (le Viseur et al., 2008), but its origin is still the subject of continuing discussion.

The *ETV6-RUNX1* chromosomal alteration is the most common aberrant genetic finding in childhood B-ALL (Pui et al., 2019a). One of the most extreme examples of genotype-phenotype relationship is the *ETV6-RUNX1* genetic alterations, as its detection is only associated with B-ALL and not to T-ALL or other types of cancer. The *ETV6-RUNX1* oncogenic lesion is found in neonatal cord blood in up to 5% of new-borns (Lausten-Thomsen et al., 2011; Schafer et al., 2018), those carrying the oncogenic lesion are the preleukemic clones. But only a few *ETV6-RUNX1* carriers will develop the disease, as the incidence of the disease in human beings is much lower than the detection of the preleukemic clone in healthy children. These findings suggest that the preleukemic clone (the *ETV6-RUNX1* positive cell) is susceptible to malignant transformation through the acquisition of supplementary secondary genetic events that will be acting as drivers of leukemogenesis. Consequently, *ETV6-RUNX1* does not give the impression to be a traditional dominant oncogene as it acts as a driver at the initial stage of the preleukemic cell appearance, in the hematopoietic stem cell subset. But later on, further drivers taking place in the already predisposed cell, the preleukemic clone, are necessary to fully transform the cell in a leukemic one and, at that time, *ETV6-RUNX1* fusion gene will not be necessary for leukemic propagation. But its role might be prompting an epigenetic priming in an uncommitted cell subset of the hematopoietic system that induces an aberrant B cell differentiation program that is later on susceptible of transformation. This has been proved in an *ETV6-RUNX1* mouse model where the fusion gene was restricted to hematopoietic stem/progenitor cells (HS/PCs) with the purpose of reveal this new possible way of action of *ETV6-RUNX1* (the Sca1-*ETV6-RUNX1* mice) (Rodriguez-Hernandez et al., 2017). This mouse model mimicked human disease quite well and settled solely pB-ALL with a low incidence, similar to human leukemias, but only after being exposed to common infections (Rodriguez-Hernandez et al., 2017). Hence, this model resembles human disease not only in incidence, phenotype and genetics features but also linking the exposure to environmental factors, infections, to the development of the disease as it has been hypothesized in humans after epidemiological studies (Greaves, 2018). Leukemic cells arising in these mice not only resemble the human phenotype but also share the genetic defects identity in ETV6-RUNX1 human leukemia (Rodriguez-Hernandez et al., 2017). As the genetic alteration is not expressed in the bulk of the B committed tumoral cells, *ETV6-RUNX1* is acting as a “hit and run” oncogene that primes the cell-of-origin (HS/PC) to a B cell program preleukemic clone that due to a second hit (exposure to infection) is fully transformed into a leukemic cell (Figure 1).

The ETV6-RUNX1 model is not the only case of B-ALL epigenetic priming (Table 1). The *BCR-ABL*^p190^ can also confer genetic susceptibility to B-ALL in a similar way to *ETV6-RUNX1* (Martin-Lorenzo et al., 2018). In this case, the restriction of *BCR-ABL*^p190^ to HS/PC also primed the cells to a preleukemic stage and afterward this genetic insult is not required anymore. In this model, the secondary genetic event is not induced by the exposure to infection, and forcing the presence of the second hit (*Pax5* deletion) from the beginning of the preleukemic clone appearance, induces cell transformation in 100% of the cases (Martin-Lorenzo et al., 2018). This suggests that the first hit (*BCR-ABL*^p190^) primes the cells but the second hit (*Pax5* deletion) is acting as the real driver of the pB-ALL. It looks as if the first genetic lesion (*ETV6-RUNX1* or *BCR-ABL*^p190^) primes the cell-of-origin in a precise cell program that makes the emergence of oncogene-specific- preleukemic clones. The specific priming of the preleukemic clone will make it be susceptible to fully transformation only by exact secondary hits. Consequently, the second insult will depend on the type of priming imposed by the oncogene to be able to fully transform the preleukemic clone into the LIC. In this sense, the *ETV6-RUNX1*-preleukemic clones are susceptible to transformation upon exposure to infections whilst *BCR-ABL*^p190^- preleukemic clones are not.

**Table 1:** Driving of leukaemia by a malignant epigenetic stem cell priming.

Genetic alteration	Target cell	Leukemia phenotype	References
*ETV6-RUNX1*	HS/PC	B-ALL	Rodriguez-Hernandez et al., 2017
*BCR-ABL^p190^*	HS/PC	B-ALL	Martin-Lorenzo et al., 2018
*BCR-ABL^p210^*	HS/PC	CML	Perez-Caro et al., 2009
*LM02* overexpression	HS/PC	T-ALL	Garcia-Ramirez et al., 2018

*HS/PC, hematopoietic stem/progenitor cells; B-ALL, B-cell acute lymphoblastic leukemia; T-ALL, T-cell acute lymphoblastic leukemia; CML, chronic myeloid leukemia.*

It has been shown that cytogenetic subtypes of ALL showed a clear correlation with methylation profile (Milani et al., 2010; Figueroa et al., 2013; Nordlund et al., 2013; Gabriel et al., 2015; Chaber et al., 2017), such signatures have been used to design DNA methylation classifiers (Nordlund et al., 2015). These observations are in line with the fact that the initial genetic insult is imposing a specific leukemic phenotype mediated by the priming of distinctive epigenetic profiles suggesting a genetic basis for the tumor specific epigenetic changes. Those epigenetic programs are also decisive for normal B cell differentiation (Martin-Subero and Oakes, 2018).

Another recent finding connecting the epigenetic priming in childhood B-ALL comes from the study of concordant BCP-ALL twins with TCF3-ZNF384 and PTPN11 mutations (Bueno et al., 2019). In this study, the cell-of-origin was identified as a stem cell or a nascent fetal progenitor. The preleukemic clone harboring the first hit, the identical TCF3-ZNF384 rearrangement shared by both twins, evolved to the leukemic stage in parallel in both patients (Bueno et al., 2019). The described acquisition of a shared hypomethylation profile in the tumor cells of this pair of monozygotic twins fits with the idea that the first hit is priming the cell-of-origin in a specific epigenetic route that depends on the initial event of transformation. In addition, the initial translocation models the cell-of-origin in such a way that favors the acquisition of secondary mutations that do not have to be identical, although they do affect the same target (D61Y and T507K activating mutations of PTPN11). Later on, leukemia evolves independently of the initial hit and secondary twin-specific mutations accumulate during tumor progression (Bueno et al., 2019). The conserved hypomethylation profile described in Bueno et al. (2019) in the leukemic cells also fits with previous findings in mouse models of epigenetic reprogramming (Vicente-Duenas et al., 2012, 2019; Green et al., 2014). As an example, restricted expression of BCR-ABLp210 to hematopoietic stem-progenitor cells (HS/PC) leads to CML *in vivo* by inducing a global hypomethylation in the stem cells (the cell-of-origin) that is maintained in the mature myeloid tumor cells (Vicente-Duenas et al., 2019).

## Epigenetic Priming in Childhood T-ALL

The oncogene *Lim Domain Only 2* (*LMO2*) is one of the most frequent drivers of childhood T-cell acute lymphoblastic leukemia (T-ALL) and accounts for about 15 to 25% of T-ALLs in children and adults (Van Vlierberghe et al., 2006; Qian et al., 2019). The intimate relationship between the aberrant expression of *LMO2* and human T-ALL development can be supported by two alternative explanations. The first one is the classical interpretation that considers that the initiating genetic alteration, the aberrant expression of *LMO2*, arises in a differentiated T-cell and has been supported by several studies (McCormack et al., 2010; Cleveland et al., 2013; Smith et al., 2014; Ruggero et al., 2016). Under these conditions, the oncogene (*LMO2*) is fundamental and necessary for the transformation and maintenance of the cell where the tumor originates (the committed-T cell). Although during the evolution of the tumor, the cells will acquire secondary alterations that will define the clinical characteristics of the disease and further decontrol its behavior. But the expression of the oncogene (*LMO2*) is necessary for all the steps of the disease. Because cell transformation arises in a differentiated cell (a T cell), this is the reason why tumor cells have such a phenotype.

Nevertheless, if we consider the epigenetic priming mechanism, we can explain the relationship between *LMO2* and T-ALL in a different way. Under this hypothesis, *LMO2* oncogene is directly capable of priming the phenotypic characteristics of the tumor in a non-committed T cell that could be an HS/PC (Figure 1). And the T-cell phenotype of the leukemic cell is the result of the priming of the cell-of-origin imposed by the aberrant expression of *LMO2*. In this sense, T-ALL is also a scenario for epigenetic priming. This second possibility has been proved in mouse models that ectopically express *LMO2* in non-T target cells (Garcia-Ramirez et al., 2018). These new T-ALL models driven by *LMO2*, reveal that the tumor T-cell personality is imposed by the oncogene instead of by the cell-of-origin phenotype (Garcia-Ramirez et al., 2018). In this model, *LMO2* operates as a “hit-and-run” oncogene. In this sense, is able to prime the hematopoietic stem/progenitor cells (HS/PCs) into a T-cell developmental program. This priming to T-ALL is affected by the vulnerability of the cancer cell-of-origin. This means that the capability of reprogramming is decreased when the oncogene is expressed in more differentiated progenitors in the hematopoietic system (Garcia-Ramirez et al., 2018). The *LMO2* genetic lesion that initiates the T-ALL process is replaceable for the subsequent progression and maintenance of the tumor. While evolved tumor cells are dependent on secondary oncogenic procedures, like *Notch1* mutations. Despite the absence of Lmo2 protein within the leukemic T-cells, these mice progress to a clonal and aggressive T-ALL (Garcia-Ramirez et al., 2018).

The reprogramming capacity of *LMO2* has been also proved as is one of the six transcription factors whose transient expression is mandatory for the reprogramming of terminally differentiated blood cells in the mouse into induced hematopoietic stem cells (Batta et al., 2014; Riddell et al., 2014). In the same direction, *LMO2* expression due to retroviral insertion and transactivation in CD34^+^ HSCs of X-SCID patients caused T-ALL but no other hematopoietic tumors (Hacein-Bey-Abina et al., 2008; Howe et al., 2008). Also, supporting this theory is the fact that epigenomic regulators are recurrently mutated in T-ALL (68% of cases). And if we focus on T-ALL driven by *LMO2*, recurrent mutations in USP7, PHF6, and EZH2 have been found (Liu et al., 2017; Qian et al., 2019).

A similar reprogramming process of transformation is conducted by the Human T Cell Lymphoma Leukemia Virus (HTLV-1) responsible for the Adult T cell Leukemia (ATL). In this regard, the virus-oncoprotein Tax is essential for the initial transformation process with pleiotropic actions in the infected cell, but upon initial infection and transformation of the cell, the oncoprotein is no longer expressed by the retrovirus. It has been described genetic and epigenetic abnormalities that replace the function of Tax and are responsible for the maintenance of the leukemic cells after the initial malignancy process imposed by Tax (Yamagishi and Watanabe, 2012). On the other hand, it has always been thought that the HTLV-1 virus preferentially infects CD4^+^CCR4^+^ T cells, and this is why ATL leukemias are developed. But, interestingly, it has been shown that HTLV-1 is able to infect hematopoietic stem cells (HSCs) and converts the precursor infected cells into specialized T cells (Furuta et al., 2017). Taken together these results, it seems that the epigenetic priming is also behind the ATL development.

## Leukemia-Epigenetic Priming Implications

The leukemia-epigenetic priming mechanism opens new possibilities to treat ALL. As epigenetic modifications can be manipulating, and somehow reinitiated, maybe in the near future it could be possible to identify them in the preleukemic clones and modify in order to interrupt the malignant priming. In this line, B-ALL cells have been reprogrammed using a DNA methyl-transferase inhibitor to an alternative lineage cell fate and making the disappearance of its malignant behavior (Bhatla et al., 2012). Also, epigenetic reprogramming has been used as a therapy to kill leukemia stem cells (Scott et al., 2016).

In any case, a better understanding of the epigenetic priming in leukemia is a prerequisite to offer and develop novel potential therapeutic approaches. To this aim, the development of preclinical models as the ones summarized in this review are unique tools to this purpose.

## Author Contributions

All authors listed have made a substantial, direct and intellectual contribution to the work, and approved it for publication.

## Conflict of Interest Statement

The authors declare that the research was conducted in the absence of any commercial or financial relationships that could be construed as a potential conflict of interest.
